# Protocol for using *MYOD1*-transduced human urine-derived cells as a predictive platform for exon skipping therapy in Duchenne muscular dystrophy

**DOI:** 10.1016/j.xpro.2025.103856

**Published:** 2025-06-06

**Authors:** Katsuhiko Kunitake, Takami Ishizuka, Eri Takeshita, Hirofumi Komaki, Yoshitsugu Aoki

**Affiliations:** 1 Department of Molecular Therapy, National Institute of Neuroscience, National Center of Neurology and Psychiatry (NCNP), Kodaira, Tokyo 187-8502, Japan; 2 Translational Medical Center, National Center of Neurology and Psychiatry (NCNP), Kodaira, Tokyo 187-8502, Japan; 3 Department of Child Neurology, National Center Hospital, National Center of Neurology and Psychiatry (NCNP), Kodaira, Tokyo 187-8551, Japan

**Keywords:** Cell Biology, Cell culture, Cell isolation, Health Sciences, Genetics, Molecular Biology, Stem Cells, Cell Differentiation

## Abstract

Antisense oligonucleotide (ASO)-based exon skipping is a splice-modulating therapy effective for Duchenne muscular dystrophy (DMD) caused by dystrophin deficiency. Here, we present a protocol for evaluating exon skipping efficacy in *MYOD1*-transduced human urine-derived cells (MYOD1-UDCs) from patients. We describe steps for isolating UDCs, selecting CD90-positive cells, inducing myogenic differentiation, and assessing the restoration of DMD mRNA and proteins after exon skipping. This platform enhances the predictability of ASO screening, promoting early-stage drug discovery and translational research in DMD.

For complete details on the use and execution of this protocol, please refer to Komaki et al.^1^

## Highlights

• Protocol to isolate UDCs from DMD patient-derived urine
• Instructions for FACS of CD90-positive UDCs, which possess high myogenic potency
• Steps for evaluating ASO efficacy in *MYOD1*-converted UDCs

## Abstract (editor highlights)

Publisher’s note: Undertaking any experimental protocol requires adherence to local institutional guidelines for laboratory safety and ethics.

## Graphical abstract

**Figure:**
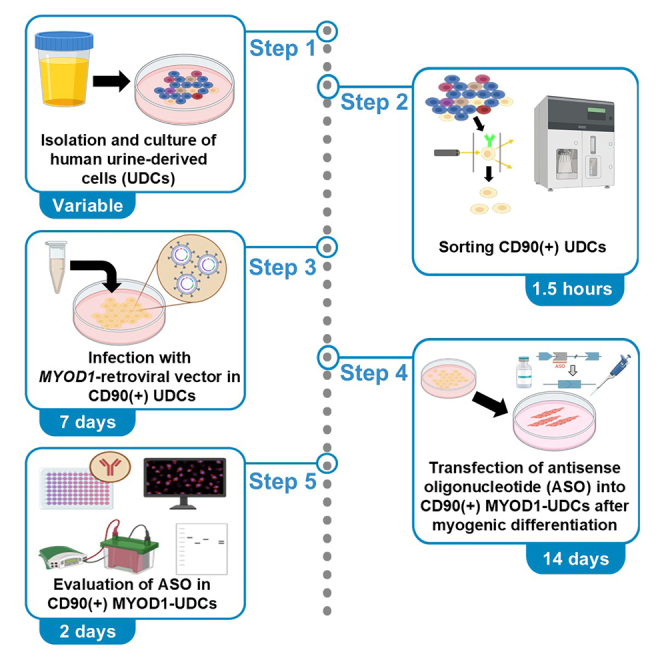

## Before you begin

Duchenne muscular dystrophy (DMD) is a progressive muscle-wasting disease caused by out-of-frame mutations in the *DMD* gene, resulting in dystrophin protein deficiency.^2^ A promising therapeutic strategy involves exon skipping using antisense oligonucleotides (ASOs), which can restore dystrophin expression by converting out-of-frame transcripts into in-frame ones.^3^ Approximately 60% of DMD patients have exon deletions clustered between exons 45 and 55, prompting development of ASOs targeting this region.^4^ Our previous clinical trial (UMIN: 000010964, NCT02081625) demonstrated that the exon 53-skipping ASO viltolarsen has good efficacy, safety, and pharmacokinetics.^5^ However, variable patient response highlighted the need for pre-treatment validation using patient-derived cells. We focused on human urine-derived cells (UDCs), which can be collected non-invasively and repeatedly.^6^^,^^7^ Incidentally, CD90 was identified as relevant to myogenic potential, being expressed in early but not mature myotubes.^8^^,^^9^^,^^10^ Accordingly, our single-cell RNA-sequencing revealed that a highly myogenic UDC subpopulation exhibited CD90.^11^ Moreover, ASO-treated CD90-positive MYOD1-UDCs from DMD patients exhibited stable high dystrophin expression.^11^ Subsequently, we conducted a Phase 1/2 trial of brogidirsen, an exon 44-skipping ASO (UMIN: 000038505, NCT04129294). After treatment with 40 mg/kg and 80 mg/kg doses for 24 weeks, we observed dose-dependent increases in dystrophin levels - 16.63% and 24.47% of normal, respectively - along with safe and favorable pharmacokinetics.^1^ MYOD1-UDCs derived from the patients also showed dose-dependent dystrophin increases *in vitro* when exposed to brogidirsen. Future clinical trials can utilize this *in vitro* assay to pharmacological evaluation or patient stratification for specific ASO drugs. Here, we provide detailed protocols for isolating UDCs from urine, inducing myotube formation, and assessing ASO-mediated exon skipping and dystrophin restoration in patient-derived cells.

### Institutional permissions

Human urine samples were acquired from DMD patients at the NCNP hospital, and this study was approved by the facility’s ethics committee. Before registration to our research, legal representatives of every patient wrote informed consent, and patients themselves provided voluntary assent. All experiments followed the Declaration of Helsinki and all Japanese local and national regulatory laws related to the study.

## Key resources table

**Table:** 

REAGENT or RESOURCE	SOURCE	IDENTIFIER
**Antibodies**		

Rabbit polyclonal anti-dystrophin (1:500)	Abcam	Cat# ab15277; RRID: AB_301813
Mouse monoclonal anti-GAPDH (1:1,000)	Millipore	Cat# MAB374; RRID: AB_2107445
Fluorescein isothiocyanate (FITC)-conjugated anti-human CD90 antibody (1:200)	BioLegend	Cat# 328107; RRID: AB_893438
Anti-mouse horseradish peroxidase-conjugated secondary antibody (1:50,000)	Amersham	Cat# NA9310-1ML; RRID: AB_772193
Anti-rabbit horseradish peroxidase-conjugated secondary antibody (1:50,000)	Amersham	Cat# NA9340-1ML; RRID: AB_772191
Alexa Fluor 594 goat anti-rabbit IgG (H+L) (1:300)	Invitrogen	Cat# A11037; RRID: AB_2534095

**Biological samples**		

Human urine-derived cells from male DMD patients under 18 years old	Patients participating in the study	N/A

**Chemicals, peptides, and recombinant proteins**		

REGM Bullet Kit	Lonza	Cat# CC-4127
High glucose Dulbecco’s modified Eagle’s medium (DMEM)	GE Healthcare	Cat# 044-29765
Tetracycline-free fetal bovine serum (FBS)	Clontech	Cat# 631106
GlutaMAX	Thermo Fisher Scientific	Cat# 35050-061
Nonessential amino acids	Thermo Fisher Scientific	Cat# 11140-050
Basic fibroblast growth factor (bFGF)	Merck Millipore	Cat# F0251-25UG
Human PDGF-AB	PeproTech	Cat# 100-00AB
Penicillin-streptomycin (10,000 U/mL)	Thermo Fisher Scientific	Cat# 15140-122
Amphotericin B	Merck Millipore	Cat# S2942
Phosphate-buffered saline (PBS) tablets, pH 7.4	Takara Bio	Cat# T9181
0.25% Trypsin-EDTA	Thermo Fisher Scientific	Cat# 25200056
CELLBANKER 1	ZENOGEN PHARMA	Cat# 11910
Hexadimethrine bromide	Sigma-Aldrich	Cat# TR-1003-G
Puromycin	Clontech	Cat# 631305
ITS liquid media supplement	Sigma-Aldrich	Cat# I3146
Endo-Porter transfection reagent	Gene Tools	Cat# 2922498000
UltraPure DNase/RNase-free distilled water	Thermo Fisher Scientific	Cat# 10977-015
QIAGEN OneStep RT-PCR kit	QIAGEN	Cat# 210212
RNeasy kit	QIAGEN	Cat# 74104
Radioimmunoprecipitation assay (RIPA) buffer	Thermo Fisher Scientific	Cat# 89901
cOmplete protease inhibitor cocktail	Roche	Cat# 4693116001
Pierce BCA protein assay kit	Thermo Fisher Scientific	Cat# 23227
NuPAGE LDS sample buffer (4×)	Invitrogen	Cat# NP0007
NuPAGE sample reducing agent (10×)	Invitrogen	Cat# NP0009
NuPAGE Tris-acetate SDS running buffer	Invitrogen	Cat# LA0041
EzFastBlot HMW	Atto	Cat# AE-1460
Methanol	Fujifilm	Cat# 131-01826
ECL Prime blocking agent	Cytiva	Cat# RPN418
ECL Prime western blotting detection reagent	Cytiva	Cat# RPN2232
Tween 20	Fujifilm	Cat# 167-11515
4% paraformaldehyde	Fujifilm	Cat# 163-20145
Triton X-100	MP Biomedicals	Cat# 9002-93-1
Goat serum	Thermo Fisher Scientific	Cat# 16-210-064
Hoarse serum	Sigma-Aldrich	Cat# H1138
Doxycycline	LKT Laboratories	Cat# D5897
DAPI solution	DOJINDO	Cat# 340-07971

**Oligonucleotides**		

*MYOD1* retroviral vector containing a Tet-on system and puromycin-resistant gene	Unique vector reported previously (H. Takizawa, et al., *Sci Rep* 2019)	N/A
Antisense oligo nucleotides for each target	Gene Tools	N/A

**Software and algorithms**		

Cell Sorter software	Sony	N/A

**Other**		

Gelatin-coated MICROPLATE 6-well with lid	IWAKI	Cat# 4810-020
Sterile disposable polystyrene bottle for collecting urine	Corning	Cat# 430281
Gelatin-coated 60 mm dish	IWAKI	Cat# 4010-020
5 mL polystyrene round-bottom tube with 40 μm cell-strainer cap	Corning	Cat# 352235
5 mL polystyrene round-bottom tube	Corning	Cat# 352058
100 μm sorting chip	Sony	Cat# LE-C3210
Sony FACS SH800S	Sony	Cat# SH800S
Gelatin-coated 100 mm dish	IWAKI	Cat# 4020-020
Collagen-coated microplate 12-well/flat bottom	IWAKI	Cat# 4815-010
Collagen-coated microplate 96-well/flat bottom	IWAKI	Cat# 4860-010
NanoDrop	Thermo Fisher Scientific	Cat# ND-ONE-W
NuPAGE 3%–8% Tris-acetate protein gels	Invitrogen	Cat# EA03785BOX
XCell SureLock mini-cell	Invitrogen	Cat# EI0001
Immobilon-P transfer membrane (PVDF)	Merck	Cat# IPVH304F0
Extra thick blot filter paper	Bio-Rad	Cat# 1703965
Semidry transfer apparatus	BIO CRAFT	Cat# BE-330
ChemiDoc MP imaging system	Bio-Rad	Cat# 170-8280J1
BZ-X810 fluorescence microscope	Keyence	Cat# BZ-800
MultiNA	Shimadzu	Cat# MCE-202
Neon transfection system	Thermo Fisher Scientific	Cat# MPK5000
Thermocycler	Thermo Fisher Scientific	Cat# 4375786

## Materials and equipment

**Table:** Growth Medium (GM)

Reagent	Final concentration	Amount
REBMedium	40.87%	212.5 mL
High glucose Dulbecco’s modified Eagle medium	40.87%	212.5 mL
Hydrocortisone in REGMedium SingleQuot Kit Supplements	0.096%	0.5 mL
EGF in REGMedium SingleQuot Kit Supplements	0.096%	0.5 mL
Epinephrine in REGMedium SingleQuot Kit Supplements	0.096%	0.5 mL
Insulin in REGMedium SingleQuot Kit Supplements	0.096%	0.5 mL
Triiodothyronine in REGMedium SingleQuot Kit Supplements	0.096%	0.5 mL
Transferrin in REGMedium SingleQuot Kit Supplements	0.096%	0.5 mL
Tetracycline-approved fetal bovine serum	14.42%	75 mL
Glutamax	0.48%	2.5 mL
Nonessential amino acids	0.48%	2.5 mL
2.5 mg/mL basic fibroblast growth factor	0.096%	0.5 mL
10 μg/mL human PDGF-AB	0.024%	0.125 mL
Penicillin/streptomycin	0.96%	5 mL
0.5 μg/mL amphotericin B	0.19%	1 mL
**Total**	**100%**	**520 mL**

Storage conditions: Store at 4°C for up to 1 month.

**Table:** Differentiation Medium (DM)

Reagent	Final concentration	Amount
High glucose Dulbecco’s modified Eagle medium	93.47%	500 mL
Glutamax	0.93%	5 mL
Horse serum	4.67%	25 mL
ITS Liquid Media Supplement	0.93%	5 mL
**Total**	**100%**	**535 mL**

Storage conditions: Store at 4°C for up to 1 month.

## Step-by-step method details

### Isolation and culture of UDCs

**Timing: 0.5 h for seeding urine and 30 days to make the cell stocks**

**Figure 1:**
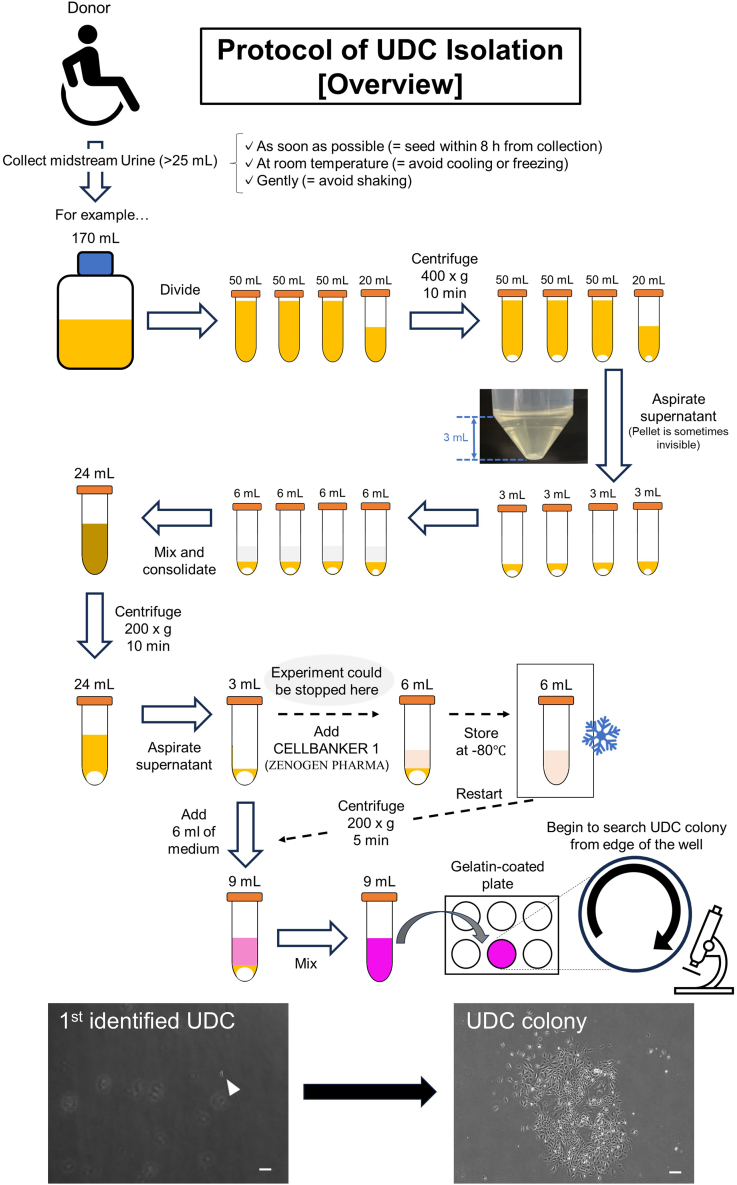
Overview of UDC-isolation from each donor Representative images of UDCs during isolation are shown. The white arrowhead in the left panel indicates a single UDC. The scale bar denotes 100 μm.

This section describes all the essential steps required to isolate and culture UDCs (Figure 1).

1. Collect >25 mL of midstream urine in a 250 mL-sized sterile disposable polystyrene bottle (Corning).

***Note:*** We understand it is difficult to collect midstream urine in some donors, including bedridden and indwelling urinary catheters. However, from the perspective of reducing the risk of bacterial contamination, midstream urine is preferable. In that context, it is recommended that urine is collected in the bottle treated with 2 mL of P/S and 400 μL of 0.5 μg/mL amphotericin B. Moreover, the urine should be seeded in plates within 8 h and stored at 15°C–30°C. Importantly, urine storage at 4°C might reduce the cell viability.

2. Dispense the total volume of collected urine into multiple 50 mL tubes and centrifuge them at 400 × *g* for 10 min at 15°C–30°C.
3. Aspirate the supernatant from each tube but, leave 3 mL, and add 3 mL of PBS (Takara Bio) to each cell suspension.

***Note:*** A fluid level of 3 mL is as high as the point around the beginning of the curve in the 50 mL tube.

4. Collect the cell suspensions from different tubes into a single 50 mL tube.

***Note:*** The cell pellet of UDCs during isolation is sometimes invisible. Therefore, leaving 3 mL of urine is essential to avoid discarding cell components.

5. Centrifuge it at 200 × *g* for 10 min at 15°C–30°C.
6. Aspirate the supernatant from each tube, but leave 3 mL.

**CRITICAL:** If you need to stop the experiment here, add 3 mL of CELLBANKER1 (Zenogen Pharma). Freeze and store the total of 6 mL liquid at −80°C. In our experience, we could restart the experiment and acquire viable UDCs even after 1 year. See Figure 1.

7. Mix 3 mL of the cell suspension with 6 mL of GM.
8. Seed 9 mL of the cell suspension mentioned above in a single well of a six-well gelatin-coated plate (IWAKI). It requires 0.5 h from steps 2 to 8.

***Note:*** Using GM supplemented with 10 μM Y-27632 as rho-associated protein kinase (ROCK) inhibitor might enhance the isolation efficiency of UDCs.^12^

9. After 72 h incubation at 37°C and 5% CO_2_, replace the medium with 2 mL of GM.
10. Change GM once every 2 days until the UDCs reach 70% confluency.

***Note:*** UDC colonies usually appear within 14 days and expand to 70% confluency by 30 days at the latest. To find the colonies using microscopy, it is better to check from the edge of the well clockwise. In our experience, 1^st^ colony of UDCs tends to appear at the edge of the well.

11. After washing the cells with 1 mL of PBS, detach them by incubating with 1 mL of 0.25% trypsin-EDTA (Thermo Scientific) for 5 min at 37°C and 5% CO_2_.
12. Mix 1 mL of GM with the cell suspensions and centrifuge them at 200 × *g* for 5 min at 15°C–30°C.
13. After aspirating the supernatant, make the cell stocks with CELLBANKER 1 (Zenogen Pharma) or pass the cells onto a 100 mm-sized gelatin-coated dish (Iwaki).

### Sorting CD90-positive UDCs

**Timing: 1.5 h**

**Figure 2:**
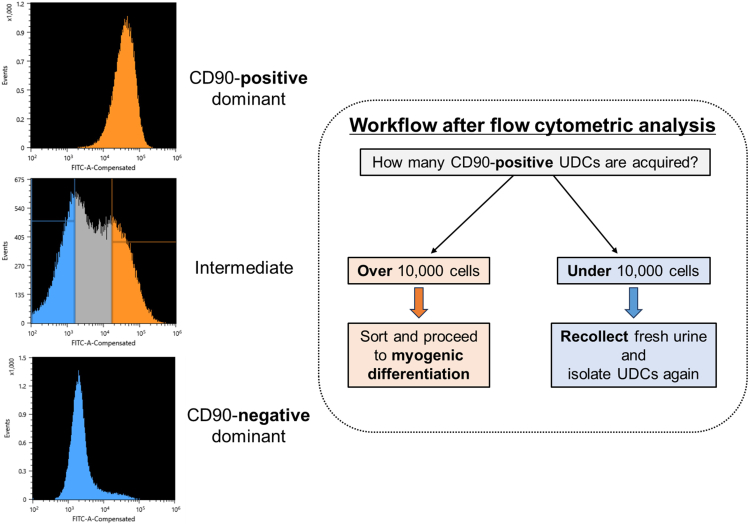
Representative flow cytometric analysis of UDCs using FITC-conjugated anti-human CD90 antibody

This section outlines the method for sorting CD90-positive subpopulations from heterogeneous UDCs (Figure 2).

14. 80% confluent cells in a 100 mm-sized gelatin-coated dish (Iwaki) should be prepared for sorting. After washing the cells with 3 mL of PBS, detach them with 3 mL of 0.25% trypsin-EDTA for 5 min incubation at 37°C and 5% CO_2_.
15. Mix 3 mL of GM with the cell suspensions and centrifuge them at 200 × *g* for 5 min.
16. Aspirate the supernatant and resuspend the cell pellet in 1 mL of PBS.
17. Mix FITC-conjugated anti-human CD90 antibody (BioLegend) with the suspension (1:200).
18. Incubate the suspension for 30 min in the dark at 4°C.
19. Centrifuge it at 200 × *g* for 5 min.
20. Aspirate the supernatant and resuspend the cell pellet in 2 mL of PBS.
21. Pass the cell suspension through a 40 μm filter cap and collect it in the tube prepared for the cell sorter.
22. Analyze the proportion of CD90-positive UDCs in the sample by fluorescence-activated cell sorting (FACS).
23. Sort CD90-positive UDCs to the collection tube if the sample contains >1.0 × 10^5^ cells of CD90-positive UDCs.

**CRITICAL:** The proportion of CD90-positive UDCs is different at every urine collection even from the same donor. If CD90-negative UDCs make up more than 90% of the total sample, the procedure should be stopped and the urine collection repeated. See Figure 2.

24. Seed CD90-positive UDCs onto a 100 mm-sized gelatin-coated dish to expand the number of cells.

### Infection with *MYOD1*-retroviral vector in CD90-positive UDCs

**Timing: 7 days**

**Figure 3:**
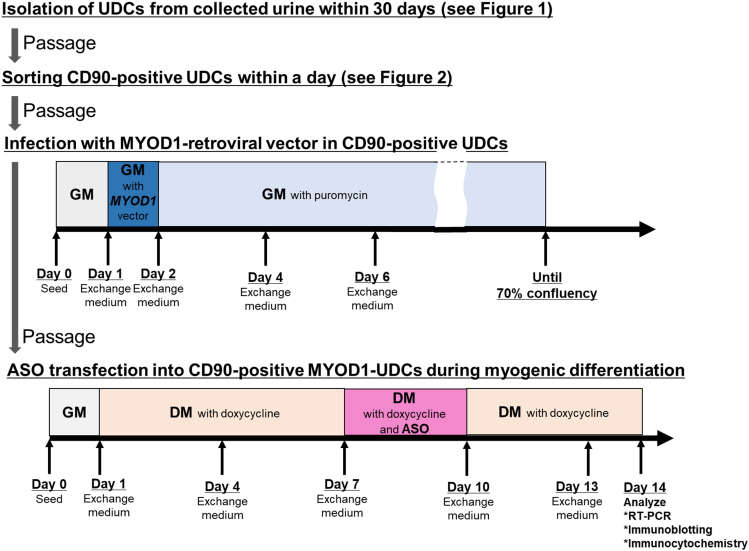
Timeline of the entire protocol from urine collection to final analysis

This section details the methods of transducing *MYOD1* gene to CD90-positive UDCs (Figure 3).

25. After passage, seed 1.6 × 10^5^ cells of CD90-positive UDCs in 10 mL of GM on a 100 mm-sized gelatin-coated dish.
26. 24 h later, replace the medium with 10 mL of fresh GM containing supernatant of *MYOD1*-inserted retroviral vector reported previously^5^ at a titer of 200 multiplicity of infection (MOI), and 8 μg/mL hexadimethrine bromide.

***Note:*** The preparation of *MYOD1*-inserted retroviral vector and related experiments requires a biosafety level (BSL)-2 laboratory. Additionally, discarded materials must be disinfected in an autoclave.

***Note:*** MOI = the copy number of retrovirus / the number of UDCs.

Granted that MOI is calculated by the equation above, 3.2 × 10^7^ copies of retrovirus are required for 200 MOI in 1.6 × 10^5^ UDCs.

27. 24 h later, replace the medium with 10 mL of fresh GM containing 10 μL of 1 mg/mL puromycin (Clontech) to select the successful *MYOD1*-transduced UDCs (MYOD1-UDCs).
28. Replace the medium with 10 mL of GM containing 10 μL of 1 mg/mL puromycin every other day until the cells expand to 70% confluency.
29. The MYOD1-UDCs could be stored at −80°C before induction of myogenic differentiation.

### ASO transfection into CD90-positive MYOD1-UDCs during myogenic differentiation

**Timing: 14 days**

This section describes all the essential steps required to transfect ASO into CD90-positive MYOD1-UDCs during myogenic differentiation (Figure 3).

30. Seed 1.0 × 10^5^ CD90-positive MYOD1-UDCs in each well of a 12-well collagen-coated plate (Iwaki). Culture in 1 mL of GM at 37°C and 5% CO_2_.

***Note:*** It is recommended that MYOD1-UDCs from each donor should be seeded on three different wells to evaluate ASO efficiency confidently.

31. 24 h later, change total GM to 1 mL of DM containing 1 μL of 1 mg/mL doxycycline (Dox).
32. 72 h later, replace total DM with 1 mL of fresh DM containing 1 μL of 1 mg/mL Dox.
33. Change 1 mL of DM containing 1 μL of 1 mg/mL Dox every 3 days.

***Note:*** It is recommended that Dox is added to DM just before each experiment.

34. On day 7, switch GM to 1 mL of DM containing 0-100 μM ASO dilutant with 6 μM Endo-Porter reagent (Gene Tools, Philomath, OR, USA) at 37°C and 5% CO_2_.

***Note:*** When we searched eSkip-Finder (https://eskip-finder.org),^13^ our in-house developed database for exon-skipping ASOs, the 0-100 μM ASO concentration range was effective in inducing exon skipping in *DMD* gene of various human muscle cells including myoblasts, MYOD1-fibroblasts and MYOD1-UDCs across nearly 9000 cases. However, in healthy donor-derived MYOD1-UDCs, exon skipping is hard to achieve even with an Endo-Porter reagent compared to DMD patient samples. Electroporation for ASO transfection could overcome this limitation. Using the Neon transfection system (Thermo Fisher Scientific) for MYOD1-UDCs cultured in GM containing 1% Dox, exon skipping could be achieved with optimized pulse conditions for UDCs as follows: 1400 V, 20 ms, and 1 pulse. Importantly, transfected MYOD1-UDCs should be cultured in GM until the cell number is recovered.

35. After the 72 h-culture with the ASO, replace the medium with 1 mL of fresh DM with 1 μL of 1 mg/mL Dox without the ASO.
36. After further 72 h, replace the medium with 1 mL of fresh DM with 1 μL of 1 mg/mL Dox without the ASO.
37. The following day (= day 14 after switching GM to DM), proceed to different types of analyses.

***Note:*** We usually calculate the sample’s fusion index in every differentiation, which is the proportion of nuclei inside MyHC-positive myotubes to total nuclei in 9 pictures randomly selected from each well. To detect clear dystrophin protein expression in the following analyses, the fusion index should be confirmed to be over 60%.

### Prediction of ASO efficacy from data of CD90-positive MYOD1-UDCs

**Timing: 1–2 days**

**Timing: 1–2 days (for step 38)**

**Timing: 2 days (for step 39)**

**Timing: 2 days (for step 40)**

This section outlines the methods of performing RT-PCR, immunoblotting and immunocytochemistry for the ASO-transfected MYOD1-UDCs.

38. Calculate exon skipping efficiency and EC_50_ based on RT-PCR products.
  a. Remove DM and wash with 1 mL PBS. Add cell lysis buffer to the differentiated CD90-positive MYOD1-UDCs, and extract total RNA using an RNeasy kit (QIAGEN).
  b. Use NanoDrop (Thermo Fisher Scientific) to measure the RNA concentration.
  c. Prepare mixtures for a one-step RT-PCR reaction (QIAGEN) following the manufacturer’s instructions.
  d. Perform PCR with the appropriate cycling conditions depending on the sample’s mutation site and designed primer sequences.
    ***Note:*** In the case of brogidirsen testing in the MYOD1-UDCs collected from DMD patients with exon 45 deletion, we designed the primers as follows:Forward; GCTCAGGTCGGATTGACATT, Reverse; GGGCAACTCTTCCACCAGTA. The following conditions were used for PCR: 1 cycle of 50°C for 30 min, 1 cycle of 95°C for 15 min, 35 cycles of 94°C for 1 min/60°C for 1 min/72°C for 1 min, 1 cycle of 72°C for 7 min, and finally hold at 4°C.
  e. Analyze the exon skipping efficiency using the MultiNA, a microchip electrophoresis apparatus (Shimadzu). The efficiency is calculated by the equation below:
    Exon skipping efficiency (%) = 100 × [skipped band molarity / (skipped band molarity + non-skipped band molarity)].
  f. Create an approximate sigmoid curve representing the relationship between ASO concentration and exon skipping. Subsequently, calculate EC_50_ as the ASO concentration equivalent to the 50% value of exon skipping (Figure 3). EC_50_ could be compared among different ASO sequences in the same donor’s MYOD1-UDCs or different donors’ MYOD1-UDC responding to the same ASO sequence.
39. Quantify restored dystrophin by immunoblotting (Figure 4).
  a. Detach the differentiated CD90-positive MYOD1-UDCs from the wells using RIPA buffer (Thermo Fisher Scientific) with cOmplete Protease Inhibitor Cocktail (Roche).
  b. After sonicating the cell lysates on ice, centrifuge at 15,000 × *g* for 15 min at 4°C.
  c. Collect the supernatant, which is expected to contain protein.
  d. Measure protein concentrations using Pierce BCA Protein Assay Kit (Thermo Fisher Scientific).
  e. Dilute 15 μg of total protein with RIPA buffer to a total volume of 10 μL in a 0.5 mL tube.
  f. Add 3 μL deionized water, 2 μL of NuPAGE Sample Reducing Agent (10×) (Invitrogen), and 5 μL of NuPAGE LDS Sample Buffer (4×) (Invitrogen) to 10 μL of the mixture stated above. Subsequently, denature the mix at 70°C for 10 min.
  g. Load 18 μL of the mixture to each lane of a NuPAGE 3-8% Tris-Acetate Protein Gel (Invitrogen), and perform electrophoresis with NuPAGE Tris-Acetate SDS Running Buffer (Invitrogen) at 150 V for 75 min in XCell SureLock Mini-Cell (Invitrogen).
  h. During the electrophoresis, activate the PVDF membrane (Merck) by methanol for 10-20 sec, and subsequently by EzFastBlot HMW (Atto) for over 10 min.
  i. Prepare four pieces of Extra Thick Blot Filter Paper (Bio-Rad), which are cut into the same size as the PVDF membrane, and activate them in the EzFastBlot HMW for over 10 min.
  j. After electrophoresis, cut the gel into the same size as the PVDF.
  k. By arranging two pieces of the Extra Thick Blot Filter Paper, the activated PVDF membrane, gel, and the other two pieces of Extra Thick Blot Filter Paper from bottom to top on the semidry transfer apparatus (BIO CRAFT), transfer at 4 mA/cm^2^ for 30 min.
  l. Block the transferred PVDF membrane with 5% ECL Prime blocking agent diluted with PBS containing 0.05% Tween 20 (PBS-T).
  m. After cutting the membrane at a height of approximately 100 kDa, incubate the upper one with primary rabbit antibody to dystrophin (1:500; Abcam, ab15277) and the lower one with mouse antibody to GAPDH (1:1,000; Millipore, MAB374) diluted in PBS-T, respectively. Subsequently, wash with PBS-T at least three times for over 1 h.
  n. Incubate the PVDF with anti-rabbit/mouse horseradish peroxidase-conjugated secondary antibody (1:50,000) diluted by PBS-T and wash with PBS-T at least three times for over 1 h.
  o. Add ECL Prime Western Blotting Detection Reagent (GE healthcare) to the PVDF and detect the target protein expression with ChemiDoc MP Imaging System (Bio-Rad). Using the software, calculate the dystrophin band’s intensity normalized to the GAPDH band’s intensity.

**Figure 4:**
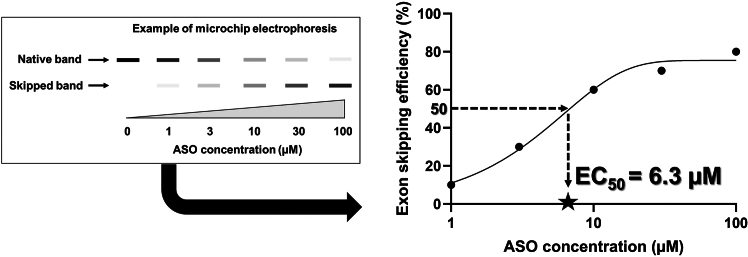
Scheme of creating a sigmoid function curve representing the relationship between ASO concentration and exon skipping efficiency to obtain the EC_50_ value
40. Observe restored dystrophin localization in the cells by immunocytochemistry (Figure 5).
  a. Fix differentiated CD90-positive MYOD1-UDCs with 4% paraformaldehyde in PBS (Fujifilm) for 10 min at 4°C after washing by PBS.
  b. Wash the cells with PBS once, and treat with 0.1% triton-X (MP Biomedicals, USA) for 5 min at 15°C–30°C.
    ***Note:*** Since UDCs are very sensitive to triton-X, the reaction time with 0.1% triton-X should not exceed 5 min.
  c. Block the cells with 10% goat serum (Thermo Fisher Scientific) diluted in PBS for 15 min at 37°C.
  d. Incubate the cells with primary rabbit antibody to dystrophin (1:500; Abcam, ab15277) in PBS for 8–16 h at 4°C.
  e. After washing the cells with PBS over three times, incubate them with a secondary antibody as Alexa Fluor 594 goat anti-rabbit IgG (1:300; Molecular Probes) in PBS for 30 min at 15°C–30°C.
  f. Wash the cells with PBS over three times, and incubate them with 1 mg/mL of DAPI (1:20,000; Dojindo) in PBS for 10 min at 15°C–30°C.
  g. Observe the cells with a BZ-X810 fluorescence microscope (Keyence, Osaka, Japan).

**Figure 5:**
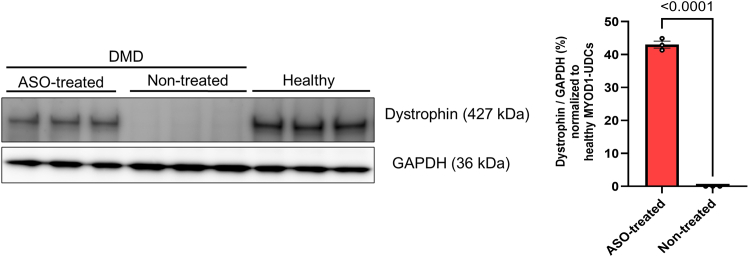
Representative images of immunoblotting in CD90-positive UDC-derived myotubes collected from an exon 50-deleted DMD patient before/after exon 51-skipping ASO treatment The data are expressed as means ± SEM. Paired t-test is used for statistical analysis, and p-value is shown. SEM = standard error of the mean.

## Expected outcomes

**Figure 6:**
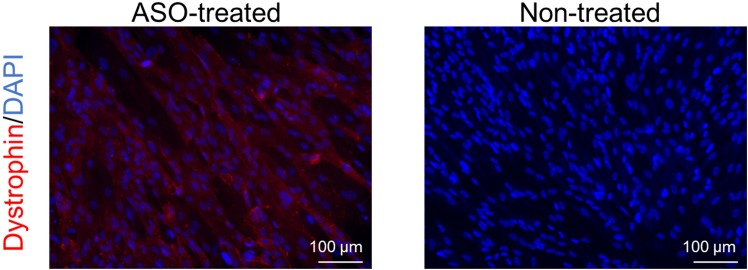
Representative images of immunocytochemistry in CD90-positive UDC-derived myotubes collected from an exon 50-deleted DMD patient before/after exon 51-skipping ASO treatment The scale bars denote 100 μm.

**Figure 7:**
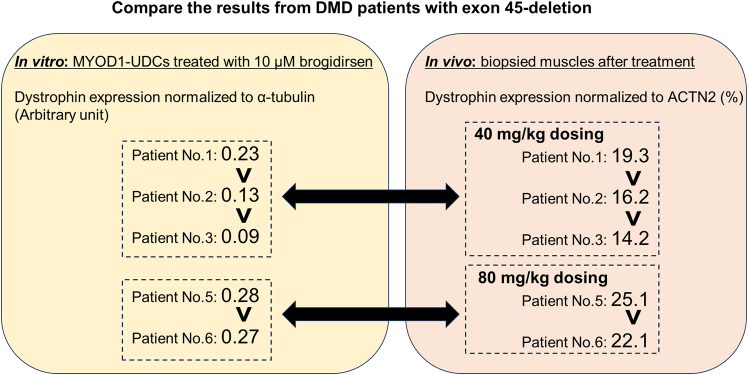
Possible correlation between *in vitro* and *in vivo* results for the case of exon 44- skipping drug, brogidirsen

After sorting UDCs with anti-CD90-antibody, CD90-positive dominant, -negative dominant, or intermediate patterns are expected to be detected (Figure 2). If more than 1.0 × 10^5^ cells from the CD90-positive subpopulations are obtained, we could proceed to *MYOD1*-induction and subsequent myogenic differentiation with ASO treatment. On day 14 of differentiation, it is anticipated that dystrophin expression will be observed in accordance with ASO efficacy by immunoblotting (Figure 5) and immunocytochemistry (Figure 6). As we summarized in Table 1, non-invasively collected MYOD1-UDCs could evaluate the dose-dependency of ASOs as well as myoblasts or MYOD1-fibroblasts, which are collected invasively from the same donors. In Figure 5, after 79% of exon 51 skipping, dystrophin expression level in CD90-positive UDC-derived myotubes collected from an exon 50-deleted DMD patient reached 43% of the protein level in those from a healthy donor. When we extracted a part of immunoblotting data treated with brogidirsen in Phase 1/2 trial,^1^ superiority or inferiority of dystrophin expression among different donors showed good correlations between MYOD1-UDCs and biopsied muscles (Figure 7). To obtain the EC_50_, defined as the half-maximal effective concentration of ASO, from RT-PCR data, at least two measured values (>50% and <50%) must bracket the 50% value at different ASO concentrations. In our experience, setting several points within 0-100 μM is usually sufficient to create an approximate sigmoid curve representing the relationship between ASO concentration and exon skipping efficiency (Figure 4). The 20.55% expression of dystrophin observed in biopsied muscle does not directly correspond to the EC_50_ value of 7.12 μM in MYOD1-UDCs.^1^ However, if a developing ASO demonstrates a lower EC_50_ in an in vitro assay using MYOD1-UDCs, we can expect a more substantial exon-skipping effect in the skeletal muscle of treated subjects. Therefore, measuring the EC_50_ of developing ASOs in MYOD1-UDCs might be a good benchmark before transitioning to clinical trials.

**Table 1:** Pros and cons of MYOD1-UDCs to MYOD1-fibroblasts and myoblasts for testing ASOs

**Pros**

• **Non-invasive: urine collection** (Skin or muscle biopsy is required for fibroblasts and myoblasts) • **Good proliferation: doubling within 24 hrs** (Doubling time is usually over 24 hrs in fibroblasts and myoblasts) • **Able to test dose-dependency as well as fibroblasts and myoblasts**

**Cons**

• **1-3 weeks are required for UDC isolation from urine** (Fibroblasts and myoblasts could be isolated within a week) • **Success rate of UDC isolation remains at approximately 85%** (Nearly 100% for fibroblasts and myoblasts) • ***MYOD1*-induction is required** (Required in fibroblasts; Not required in myoblasts)

## Quantification and statistical analysis

Significant differences in exon-skipping efficiency or dystrophin expression level between ASO-treated and non-treated samples were determined using the paired t-test (Figure 5). We considered the P value < 0.05 as statistically significant.

## Limitations

**Figure 8:**
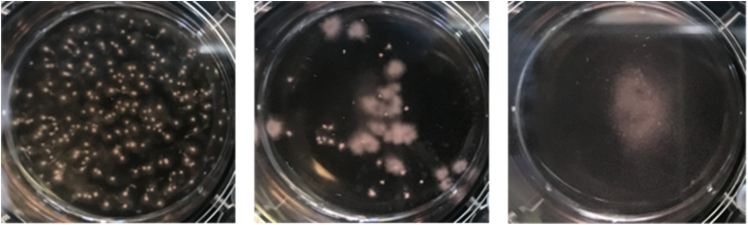
Representative images of bacterial contamination during UDC isolation

Bacterial contamination is a significant issue during UDC isolation (Figure 8). The risks for contamination include female, elderly, bedridden, and indwelling urinary catheters. However, this could be prevented by pre-treatment with the antibiotics stated above. Moreover, some of the DMD patients have comorbidities, including intellectual disability (up to 22% of patients), autism (up to 6%), and attention deficit disorders (up to 18%) due to disturbance of brain dystrophin-gene products.^14^ Therefore, we sometimes experience difficulty acquiring urine samples from such cases. Using DMD-derived MYOD1-UDCs, we could evaluate the responsiveness of donors' myotubes to each ASO or dose dependency of the drug before administration.

## Troubleshooting

### Problem 1

No UDCs can be obtained from urine samples (related to Steps 1-13).

### Potential solution

Request the clinician to provide an additional urine sample from the same patient. We sometimes experience no appearance of UDC colonies from the well without bacterial contamination. In such a case, we should try urine collection more than twice on the same day. Additionally, it might be essential to avoid collecting urine first in the morning. In our experience, we could not isolate UDCs from the urine at that hour.

### Problem 2

Few CD90-positive UDCs can be obtained from urine samples (related to Steps 14-24).

### Potential solution

At this point, we could not control the percentage of CD90-positive cells in obtained UDCs. Communicate with the clinician to request an additional urine sample from the same patient.

### Problem 3

After puromycin selection, only a few MYOD1-UDCs can be expanded to obtain sufficient cell numbers to proceed to myogenic differentiation (related to Steps 25-29).

### Potential solution

Theoretically, MYOD1-UDCs in this study are resistant to puromycin due to the inserted *MYOD1*-vector. Therefore, adding puromycin every other day is possible in most cases. However, in some cases, cells are too sensitive to puromycin and the cell viability decreases by repeated exposure to puromycin. In such a case, reducing the time of exchanging the medium containing puromycin to only once a week may overcome the situation.

### Problem 4

Unclear dystrophin expression can be obtained in immunoblotting or immunocytochemistry due to immature myotubes (< 60% of fusion index) after the myogenic differentiation (related to Steps 30-40).

### Potential solution

The efficacy of the myogenic differentiation highly depends on the quantity of the initial number of UDCs just before initiation of the differentiation. In cases where only a few numbers of cells are seeded, we should stop the culture and retry to seed a sufficient number of cells.

### Problem 5

Low exon skipping and dystrophin restoration can be obtained after ASO transfection with Endo-Porter reagent (related to Steps 30-40).

### Potential solution

First, we should try electroporation to test if the problem is led by low ASO transfection or non-optimized ASO sequence or length. If the electroporation works and exon skipping efficiency is improved, replacement of the Endo-Porter reagent with another one is desired. However, in our experience, we always successfully transfect various types of 0-100 μM ASOs, including phosphorodiamidate morpholino oligomer (PMO), 2′-*O*-methyl oligonucleotide (2OMe) and peptide-conjugated PMO (PPMO) with Endo-Porter reagent in the case of DMD patient-derived MYOD1-UDCs. If the electroporation does not work and exon skipping efficiency is still low, we should optimize the ASO sequence or length. In the case of PMO and 2OMe, we could use eSkip-Finder (https://eskip-finder.org)^13^ to predict numerous patterns of novel ASO sequences to achieve effective exon skipping based on a machine learning model trained by experimental data.

## Resource availability

### Lead contact

Further information and requests for resources and reagents should be directed to and will be fulfilled by the lead contact, Yoshitsugu Aoki (tsugu56@ncnp.go.jp).

### Technical contact

Technical questions on executing this protocol should be directed to and will be answered by the technical contact, Katsuhiko Kunitake (kunitake-k@ncnp.go.jp).

### Materials availability

Unique reagents generated in this study are available from the lead contact with a completed materials transfer agreement.

### Data and code availability

This paper does not contain any custom computer codes.

## Acknowledgments

We are grateful to the study participants and their families for their valuable contributions. This work was supported by the 10.13039/501100000646Japan Society for the Promotion of Science Grants-in-Aid for Scientific Research (B) (grant number: 23K21412 to Y.A.), as well as by the Grants-in-Aid for Research on Nervous and Mental Disorders (grant number: 5-7 to Y.A.).

## Author contributions

Conceptualization, K.K. and Y.A.; methodology, K.K.; investigation, H.K., E.T., and T.I.; visualization, K.K.; funding acquisition, Y.A.; project administration, Y.A.; supervision, Y.A.; writing – original draft, K.K. and Y.A.; writing – review and editing, H.K., E.T., and T.I.

## Declaration of interests

H.K. received consulting fees from Nippon Shinyaku for work related to this study and from Biogen, Novartis, Sanofi, Sarepta, Chugai, Daiichi Sankyo, Kaneka, and Astellas for work unrelated to this study. H.K. received grant funding from Nippon Shinyaku for work related to this study and from Sanofi, Chugai, PTC Therapeutics, Taiho, and Pfizer for work unrelated to this study. E.T. received grant funding from Nippon Shinyaku for work related to this study and from Daiichi Sankyo, Taiho, and Takeda for work unrelated to this study. Y.A. received grant funding from Nippon Shinyaku for work associated with this study and from Shionogi, Takeda, and Kaneka for work unrelated to this study.
